# Phylogeography and biological characterization of H12N2 virus isolated from whooper swan in Central China

**DOI:** 10.3389/fmicb.2024.1536876

**Published:** 2025-01-09

**Authors:** Pengfei Ren, Zhen Gao, Xing Li, Jiao Tang, Pei Li, Zhonglin Huang, Jinchi Guo, Pengfei Cui, Lin Jin, Junping Li, Libin Liang

**Affiliations:** ^1^College of Veterinary Medicine, Shanxi Agricultural University, Jinzhong, China; ^2^State Key Laboratory for Animal Disease Control and Prevention, Harbin Veterinary Research Institute, Chinese Academy of Agricultural Sciences, Harbin, China

**Keywords:** avian influenza viruses, whooper swan, H12N2, evolution, biological characterization

## Abstract

Wild birds and waterfowl serve as the natural reservoirs of avian influenza viruses (AIVs). When AIVs originating from wild birds cross species barriers to infect mammals or humans, they pose a significant threat to public health. The H12 subtype of AIVs primarily circulates in wild birds, with relatively few isolates reported worldwide, and the evolutionary and biological characteristics of H12 subtype AIVs remain largely unknown. In this study, we analyzed the spatiotemporal distribution of H12 subtype AIVs worldwide and conducted a comprehensive investigation into the evolutionary and biological characteristics of an H12N2 virus isolated from a whooper swan in Central China. Phylogenetic analysis revealed that the H12N2 isolate belongs to the Eurasian lineage, with its HA gene likely originating from a duck-derived H12N5 virus and its NA gene potentially derived from an H9N2 virus, indicating that it is a complex reassorted virus. Animal experiments in domestic ducks and chickens demonstrated that the virus replicates at low levels in the respiratory tract of poultry and exhibits moderate horizontal transmission in ducks. However, it is capable of efficient horizontal transmission in chickens. Mouse infection experiments revealed that the virus could be detected in the nasal turbinates and lungs of mice, indicating that the H12N2 virus can infect mice without prior adaptation. *In vitro* studies revealed that the virus replicates efficiently in MDCK cells, with significantly higher titers than those in DF1 cells. These findings, combined with the mouse infection results, suggest that the H12N2 virus poses a potential risk of mammalian infection. This study provides valuable insights regarding the characteristics of the H12N2 virus and highlights the importance of ongoing surveillance and risk assessment of AIVs originating from wild birds.

## 1 Introduction

Avian influenza viruses (AIVs) are members of the Orthomyxoviridae family and are categorized into multiple subtypes. All 16 hemagglutinin (HA) and 9 neuraminidase (NA) subtypes have been identified in wild birds, particularly among wild waterfowl and shorebirds (Gass et al., 2022). To date, several subtypes originating from wild birds or poultry, primarily H3N8, H5Nx, H7N9, H9N2, H10N3, and H10N8, have resulted in substantial economic losses in the poultry industry and pose an increasing threat to human health (Cui et al., 2021; Gu et al., 2022; Guo et al., 2021; Liu et al., 2021; Nishikimi et al., 2022; Zhang et al., 2014). Long-distance migratory birds play critical roles in the ecology and evolution of AIVs and are regarded as natural reservoirs for these viruses (Liu et al., 2021; Zhang et al., 2023). The movement of wild birds facilitates the dispersal of AIVs along their migratory routes, potentially linking viral pools and contributing to the rapid emergence of novel strains in both wild birds and other hosts. Although AIVs are predominantly found in wild birds, an increasing number of these viruses have demonstrated the ability to cross interspecies barriers and potentially infect mammals, posing a significant challenge to public health. Therefore, understanding the prevalence of AIVs in wild birds is essential for effective risk assessment and preparedness against future outbreaks.

H12 viruses are primarily found in migratory birds and have not been detected in poultry or mammals. The first H12N2 subtype was isolated from mallards in Wisconsin in 1977 (Liu et al., 2020). As of November 22, 2024, only 30 H12N2 subtype viruses have been recorded in the GISAID EpiFlu and NCBI Influenza Virus Resource databases. Several studies have reported the isolation of H12 viruses from migratory birds and conducted genetic and phylogenetic analyses (Bui et al., 2015; Sharshov et al., 2019; Tang et al., 2020; Wan et al., 2022; Wang et al., 2024; Wille et al., 2018). However, the characteristics of H12N2 subtypes, including their ecology, phylogeography, and replication ability in poultry and mammals, remain largely unknown. In this study, we isolated a novel H12N2 virus from the fecal droppings of wild swan in Shanxi Province, Central China. Based on the uncertainties surrounding H12 AIVs, we conducted a comprehensive investigation into the ecology and evolutionary dynamics of H12N2 viruses. Specifically, our study evaluated the infection and replication capabilities of whooper swan-derived H12N2 viruses in both poultry and mammalian hosts. The findings of this research will enhance the understanding of the evolutionary paths and potential infection risks associated with these rare H12N2 viruses.

## 2 Materials and methods

### 2.1 Sample collection and virus identification

From November 2023 to February 2024, during the winter migration season of whooper swans, we collected a total of 945 fresh fecal samples from whooper swans in Pinglu Wetland, Shanxi Province (34° 49′ 51″ N, 111° 8′ 53″ E). Each fresh fecal drop was collected and placed into 2 mL of PBS supplemented with penicillin and streptomycin. The samples were vortexed, centrifuged, and inoculated into 10-day-old chicken embryos for virus isolation in an ABSL-2 laboratory at Shanxi Agricultural University. The allantoic fluid was harvested after 72 h and tested via a hemagglutination (HA) assay. The HA subtype was determined via the HI test, whereas the NA subtype was identified via PCR and sequencing as described previously (Liang et al., 2024). The virus A/swan/Shanxi/2143/2023 (H12N2) used in this study was isolated from fresh fecal samples of whooper swans collected in Shanxi Province.

### 2.2 Genetic and molecular analysis

RNA from the H12N2 virus was extracted using TRIzol reagent, followed by the synthesis of complementary DNA (cDNA) via reverse transcription with the Uni12 primer (AGCAAAAGCAGG). The eight segments of the H12N2 virus were amplified via PCR using specific primers. Sequencing was conducted by Sangon Biotech Co., Ltd. (Shanghai, China). The nucleotide sequence data were subsequently edited using the SeqMan program (DNAstar, v7.1.0). Molecular markers for each segment were identified using the MegAlign program (DNAstar, v7.1.0).

### 2.3 Data acquisition and phylogenetic analysis

Markov chain Monte Carlo (MCMC) trees with molecular clocks of the HA and NA genes of H12N2 viruses and their internal genes (PB2, PB1, PA, NP, M, and NS) were constructed using BEAST (v1.10.4) (Suchard et al., 2018). Briefly, the sequences were aligned via MAFFT (v7.453) (Katoh and Standley, 2013), and duplicate or highly similar sequences were removed using BioAider (v1.423) (Zhou et al., 2020). The model of nucleotide substitution was selected in ModelFinder of IQ-tree, and a relaxed log-normal clock was set in the BEAUti program. The best-fitting nucleotide models GTR + F + I + R3 for the HA data and TVM + F + G4 for the NA data were selected. The MCMC chain was executed for 20 million steps and sampled at 10,000-step intervals to generate a BEAST file. Phylogenetic trees were constructed for the six internal genes (PB2, PB1, PA, NP, M and NS) using the same methodology, with the best model used for each gene. Tracer (v1.7.1) was used to ascertain whether the parameters converged (effective sample size values ≥200). A target tree was obtained by selecting the tree with the largest posterior probability with a 10% burn-in using the Tree Annotator program.

### 2.4 *In vitro* growth kinetics

The growth kinetics of the H12N2 virus were determined in Madin–Darby canine kidney (MDCK) cells and chicken embryo fibroblast (DF1) cells. Briefly, confluent monolayers of cells were infected with H12N2 virus (10^4^ EID_50_/well) and incubated at 37°C. The cell supernatants were collected at 12, 24, 36, and 48 h post infection (h.p.i.), the viral titers were determined in chicken embryos, expressed as 50% egg infectious dose (EID₅₀). The experiments were performed in triplicate.

### 2.5 Duck study

Three five-week-old ducks were inoculated intranasally with 10^6^ EID_50_ of the H12N2 virus in a volume of 200 μL. On day 3 post infection (p.i.), the ducks were euthanized, and tissue samples, including trachea, lung, pancreas, liver, spleen, kidney, intestine, brain, and bursa of Fabricius, were collected for viral titration in chicken eggs. To evaluate the transmission of the virus in ducks, three ducks were inoculated intranasally with 10^6^ EID_50_ of the virus in a volume of 200 μL. An additional three naive ducks were placed in the same isolator at 24 h.p.i. to serve as the contact group. Oropharyngeal and cloacal swabs were collected from both the inoculated and contact ducks on days 1, 3, 5, 7, 9, and 11 p.i. Viral titers from the swabs were determined via titration in chicken embryos. The sera of the inoculated and contact ducks were collected on days 10, 15, and 21 p.i., and the antibody titers were assessed via the hemagglutination inhibition (HI) assay.

### 2.6 Chicken study

Specific pathogen-free (SPF) chickens (5 weeks old) were purchased from Beijing Boehringer Ingelheim Vital Biotech Co., Ltd. (Beijing, China). Three chickens were inoculated intranasally with 10^6^ EID_50_ of the H12N2 virus in a volume of 200 μL. On day 3 p.i., the chickens were euthanized, and tissue samples, including trachea, lung, pancreas, liver, spleen, kidney, intestine, brain, and bursa of Fabricius, were collected for viral titration in chicken embryos. To evaluate the transmission of the virus in chickens, three chickens were inoculated intranasally with 10^6^ EID_50_ of the virus in a volume of 200 μL. An additional three naive chickens were placed in the same isolator at 24 h.p.i. to serve as the contact group. Oropharyngeal and cloacal swabs were collected from both the inoculated and contact chickens on days 1, 3, 5, 7, 9, and 11 p.i. Viral titers from the swabs were determined through titration in chicken embryos. The sera of the inoculated and contact chickens were collected on days 10, 15, and 21 p.i., and the antibody titers were assessed via the HI assay.

### 2.7 Mouse study

Five-week-old BALB/c mice were obtained from Vital River Laboratory (Beijing. China). To evaluate the replicability of the virus in mice, groups of eleven BALB/c mice were mildly anesthetized with CO_2_ and inoculated intranasally with 10^6^ EID_50_ H12N2 virus in a volume of 50 μL. Three mice were euthanized on days 3 and 5 p.i., and the brain, nasal turbinate, spleen, kidney, and lung were collected and titrated in chicken embryos. A portion of the lung tissue was collected for histopathological observation. The remaining five mice were continuously monitored for body weight changes and survival for 14 days. Five mice inoculated with PBS were used as the control group to observe body weight changes.

## 3 Results

### 3.1 Prevalence of H12 subtype viruses in wild birds

**Figure 1:**
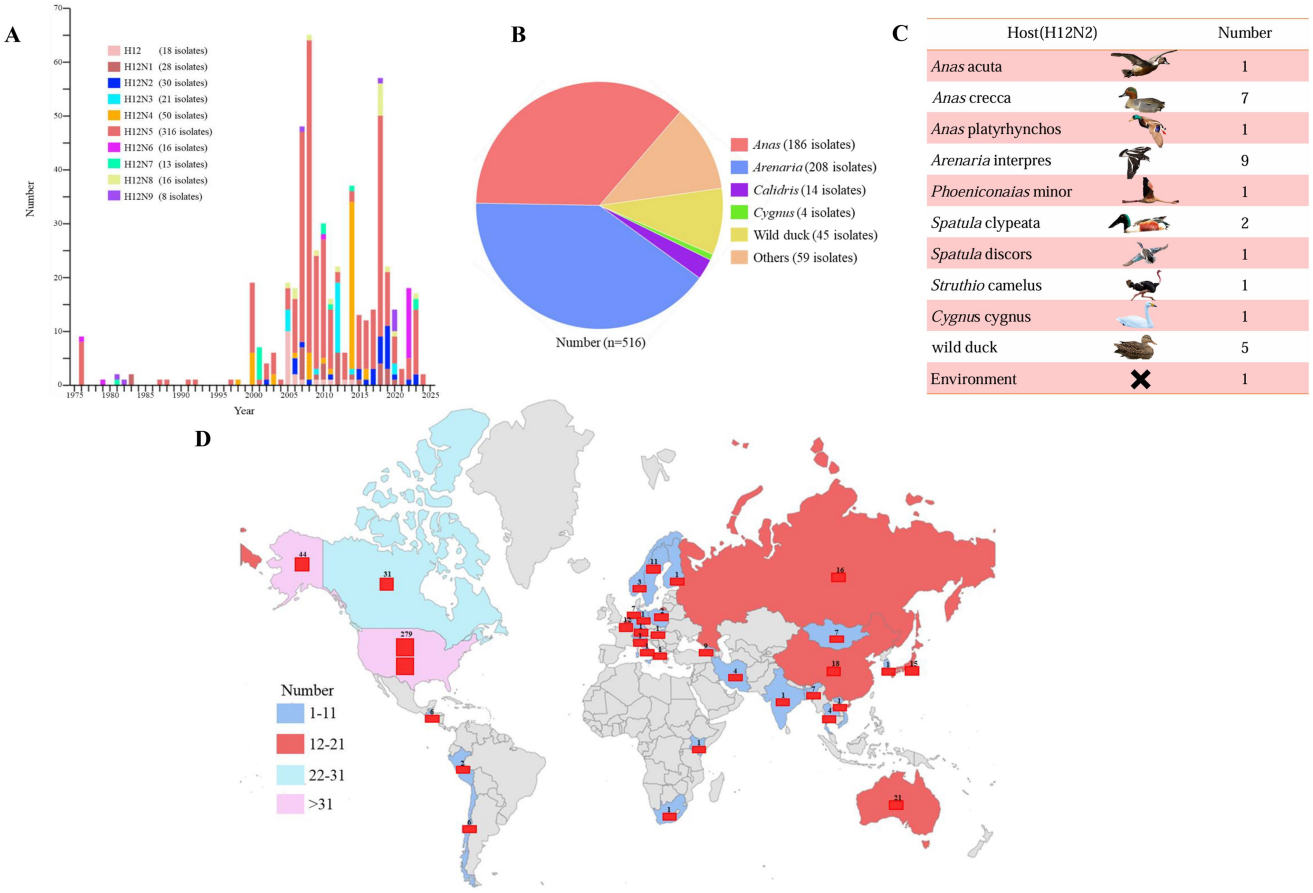
Spatiotemporal distribution of H12 subtype avian influenza viruses on a global scale. **(A)** The number of H12 subtype avian influenza viruses isolated each year, as recorded in influenza virus databases, is shown on the basis of the year of isolation. **(B)** The hosts of H12Nx viruses are summarized on the basis of sequence information obtained from GISAID and GenBank. **(C)** The host species distribution and frequency of H12N2 isolates recorded in influenza virus databases are shown. **(D)** The global spatial distribution of H12 subtype avian influenza viruses is shown. “x” in H12Nx represents the unidentified subtype of NA genes in the viruses found within the databases. All sequences were downloaded from the GISAID and GenBank databases, with data updated as of November 22, 2024.

H12 influenza viruses have been detected in wild birds since 1975; however, the epidemiology and ecology of H12 viruses remain poorly understood. To elucidate the epidemiology of H12 AIVs, we categorized the available sequence data of H12Nx (N1–N9) viruses from the GISAID and NCBI databases. After excluding duplicated and highly similar entries, we identified a total of 516 H12Nx isolates up to November 22, 2024. Statistical classification of H12Nx virus subtypes isolated between 1975 and 2024 revealed nine distinct combinations of HA and NA subtypes. H12N5 (*n* = 316) was identified as the predominant subtype, whereas H12N2 (*n* = 30) was relatively less common. The other identified subtypes included H12N1 (*n* = 28), H12N3 (*n* = 21), H12N4 (*n* = 50), H12N6 (*n* = 16), H12N7 (*n* = 13), H12N8 (*n* = 16), and H12N9 (*n* = 8) (Figure 1A). Notably, H12 viruses were highly prevalent among *Anas* (*n* = 186) and *Arenaria* (*n* = 208) (Figure 1B). Moreover, H12N2 viruses have been identified in various wild bird hosts (Figure 1C). Geographically, H12 isolates were predominantly found in North America (*n* = 354), followed by Asia (*n* = 67), Europe (*n* = 58), Australia (*n* = 21), South America (*n* = 8), Central America (*n* = 6) and South Africa (*n* = 2) (Figure 1D). These data not only enhance our understanding of H12 virus epidemiology but also facilitate improved monitoring of wild bird populations.

### 3.2 Isolation of an H12N2 virus in Central China

The Yellow River Wetland of Pinglu (Shanxi Province) is located along the East Asian–Australasian (EA) flyway of migratory birds. In recent years, approximately 8,000 whooper swans (*Cygnus cygnus*) have been observed wintering in this area each year. We conducted surveillance for AIVs from November 2023 to February 2024 in the Yellow River Wetland of Pinglu. A total of 945 fecal samples were collected and inoculated into embryonated chicken eggs. From these samples, one H12N2 virus was successfully isolated and designated A/swan/Shanxi/2143/2023 (H12N2), abbreviated as SX/2143. The entire genome of the virus was sequenced, and the sequence data were submitted to the GISAID database (accession number: EPI3408601-EPI3408608).

### 3.3 Molecular characteristics of H12N2 virus

On the basis of the complete genomic sequence of the SX/2143 virus, we analyzed the molecular characteristics of key amino acid sites in its viral proteins. These results indicate that the amino acid motif PQVQNR↓GLF is present at the cleavage site of the HA protein, reflecting typical features associated with low pathogenicity in poultry. Several amino acid substitutions were observed in the identified strain SX/2143, including PB1-D3V, H436Y and D622G, PA-A515T, M1-N30D, T215A, NS1-I106M, and C138F. These substitutions suggested that the H12 virus may have acquired a certain degree of adaptability to mammalian hosts. Additionally, the mammalian host adaptive mutations PB2-E627K and D701N were not detected in SX/2143 (Table 1).

**Table 1:** Molecular characteristics of the SX/2143 (H12N2) virus in this study.

Protein	Amino acid position	Virus name
		SX/2143
PB2	I147T	I
	A588V	A
	E627K	E
	D701N	D
PB1	D3V	V
	H436Y	Y
	D622G	G
PA	T97I	T
	A515T	T
HA	Cleavage site	PQVQNR↓GLF
	Q226L	Q
	G228S	G
M1	N30D	D
	T215A	A
NS1	P42S	A
	I106M	M
	C138F	F

### 3.4 Evolution and genetic diversity of the H12N2 virus

**Figure 2:**
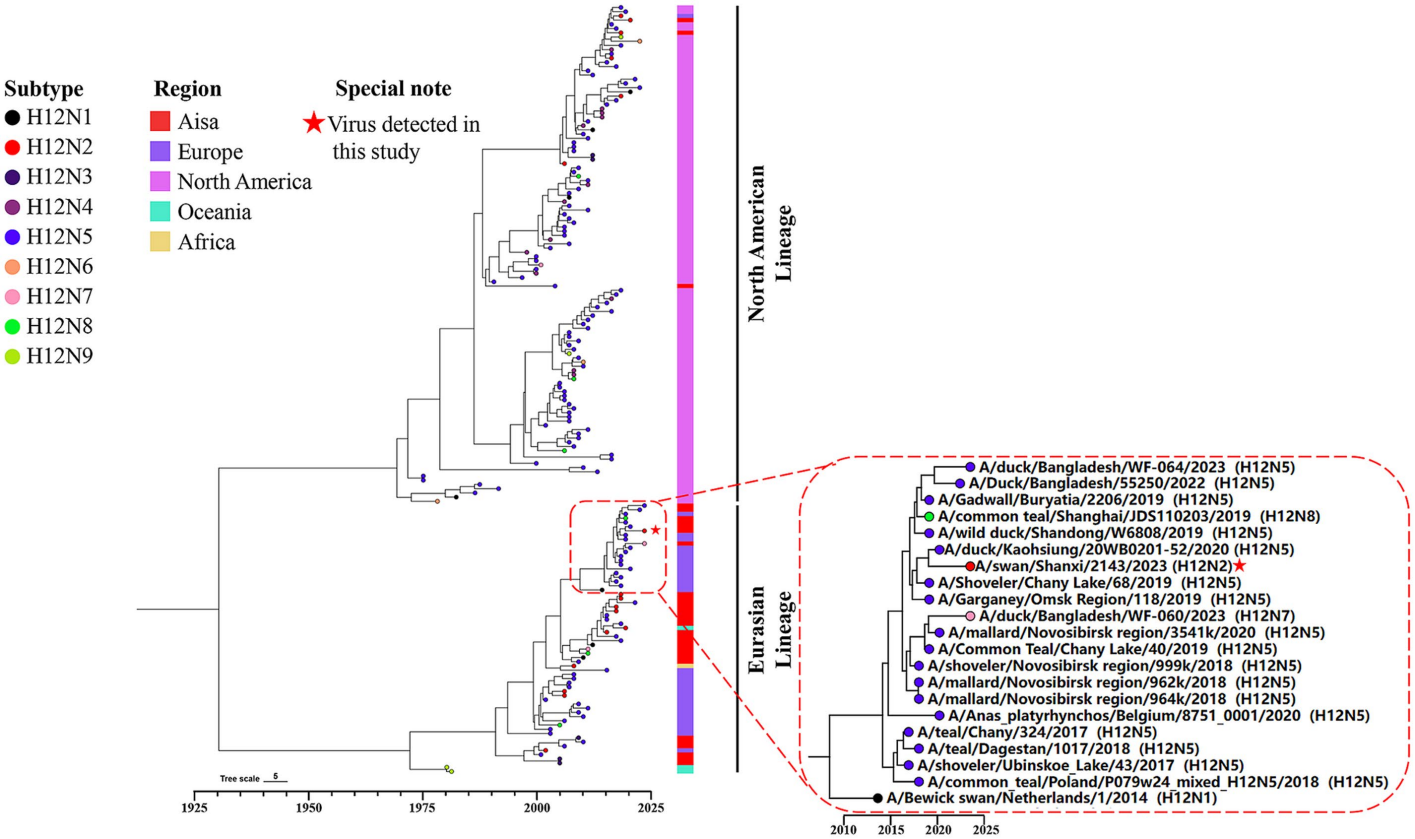
Bayesian time-resolved phylogenetic tree of the HA genes of H12 viruses. The tip points are colored according to the subtype combination (H12N1-H12N9). The collection regions of these viruses are indicated following the tip points, and the H12 viruses were collected between 1976 and 2024 (*n* = 182). The dashed box represents a group within the Eurasian lineage of the HA gene. The H12N2 virus isolated in this study is located within this group and has been highlighted with a special note.

**Figure 3:**
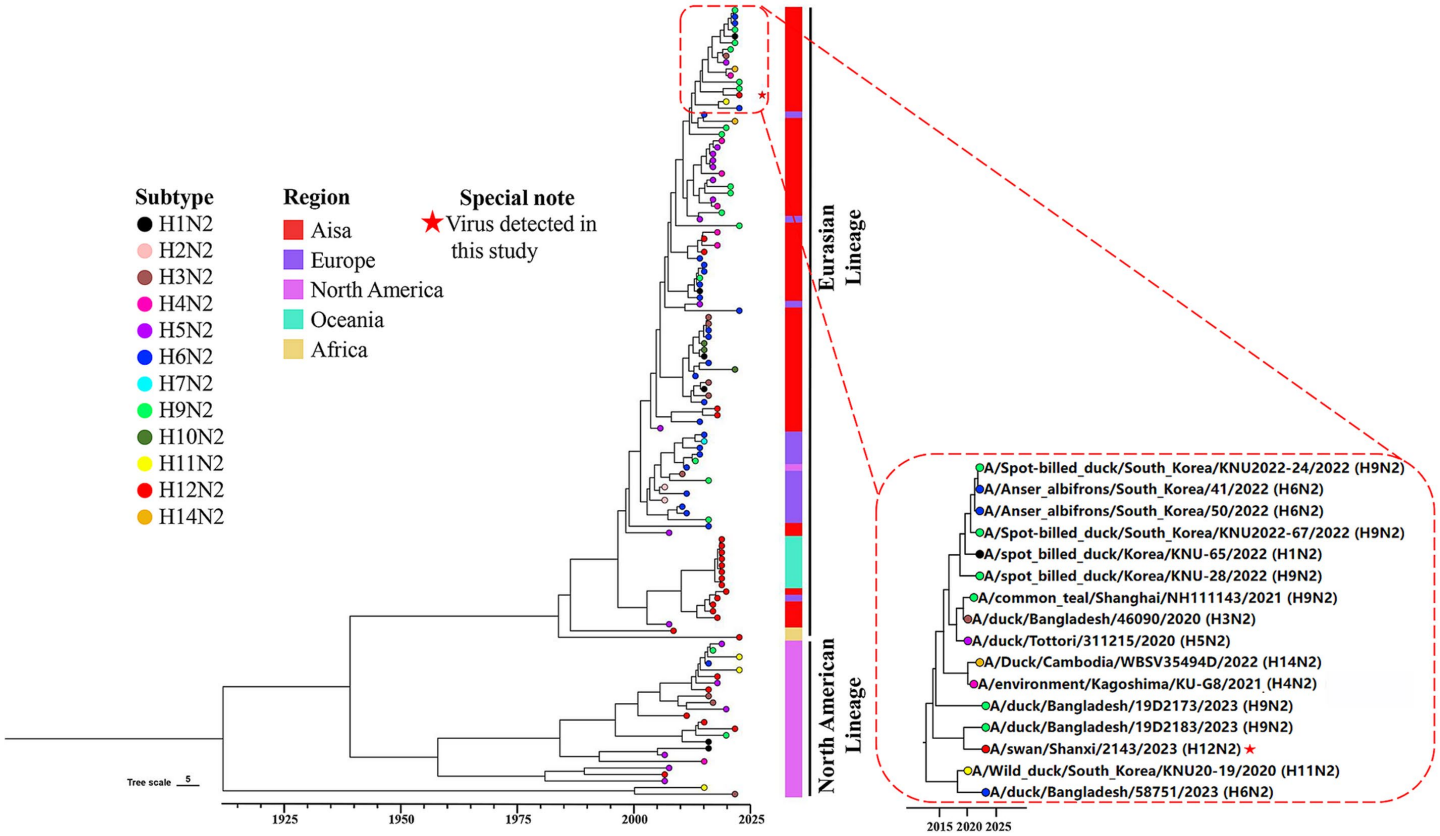
Phylogenetic diversity of the N2 genes. A maximum clade credibility (MCC) tree constructed for the NA (N2) gene (*n* = 121), encompassing various HxN2 subtypes, is shown. Different HxN2 subtype combinations are indicated by tip points in distinct colors. The isolation locations of the strains are distinguished by additional color coding. The strain isolated in this study is marked with a red five-pointed star.

To investigate the genetic evolution of the H12N2 virus isolated from the whooper swan in this study, we performed an evolutionary analysis of the HA and NA genes. A phylogenetic tree for the SX/2143 virus was constructed using reference sequences retrieved from the public influenza virus database. As shown in Figure 2, the H12 subtype HA gene can be divided into two lineages: the North American lineage and the Eurasian lineage. Most of the lineage types of the isolated strains are associated with their geographical regions; however, there were exceptions, such as certain Asian isolates whose HA genes belong to the North American lineage (Figure 2). The HA gene of SX/2143 isolated in this study belongs to the Eurasian lineage and is closely related to the H12N5 strain isolated from a duck in the Taiwan region (Figure 2). The nucleotide homology with the isolate exhibiting the highest homology reached 98.23% (Supplementary Table S1). Phylogenetic analysis of the NA genes of HxN2 subtypes revealed that the N2 genes can also be divided into North American and Eurasian lineages, with the N2 genes of the H12N2 virus forming several distinct subbranches. Notably, the N2 gene of the SX/2143 strain isolated in this study was most closely related to the N2 gene of a duck-derived H9N2 strain, suggesting that the N2 gene may have originated from the H9N2 virus (Figure 3). This relationship is supported by a nucleotide identity of 98.44%, with the isolate showing the highest homology (Supplementary Table S1).

We further constructed a phylogenetic tree of the internal genes (PB2, PB1, PA, NP, M and NS) of SX/2143 to analyze the evolutionary process and potential origins of the H12N2 strain. The PB2 and PB1 genes were most closely related to the PB2 and PB1 genes of H3N8 strains isolated from the environment or from wild birds, respectively (Supplementary Figures S1A,B). The PA gene of the H12N2 virus was likely derived from the PA gene of waterfowl H2N3 strains (Supplementary Figure S1C). The NP gene is closely related to the H8 subtype strain derived from ducks, whereas the M gene shows the highest similarity to the M gene of H4N6 viruses isolated from mallards in China (Supplementary Figures S1D,E). The NS gene had the closest relationship with H1N1 strains from wild birds (Supplementary Figure S1F). The results indicated that the internal genes of the H12N2 virus have a complex origin. The H12N2 virus may have emerged through intricate reassortment events between H12 subtype viruses and other influenza virus subtypes carried by wild birds, either during their circulation among wild bird populations or through the migration of these birds.

### 3.5 Replication and transmission of the H12N2 virus in ducks and chickens

**Figure 4:**
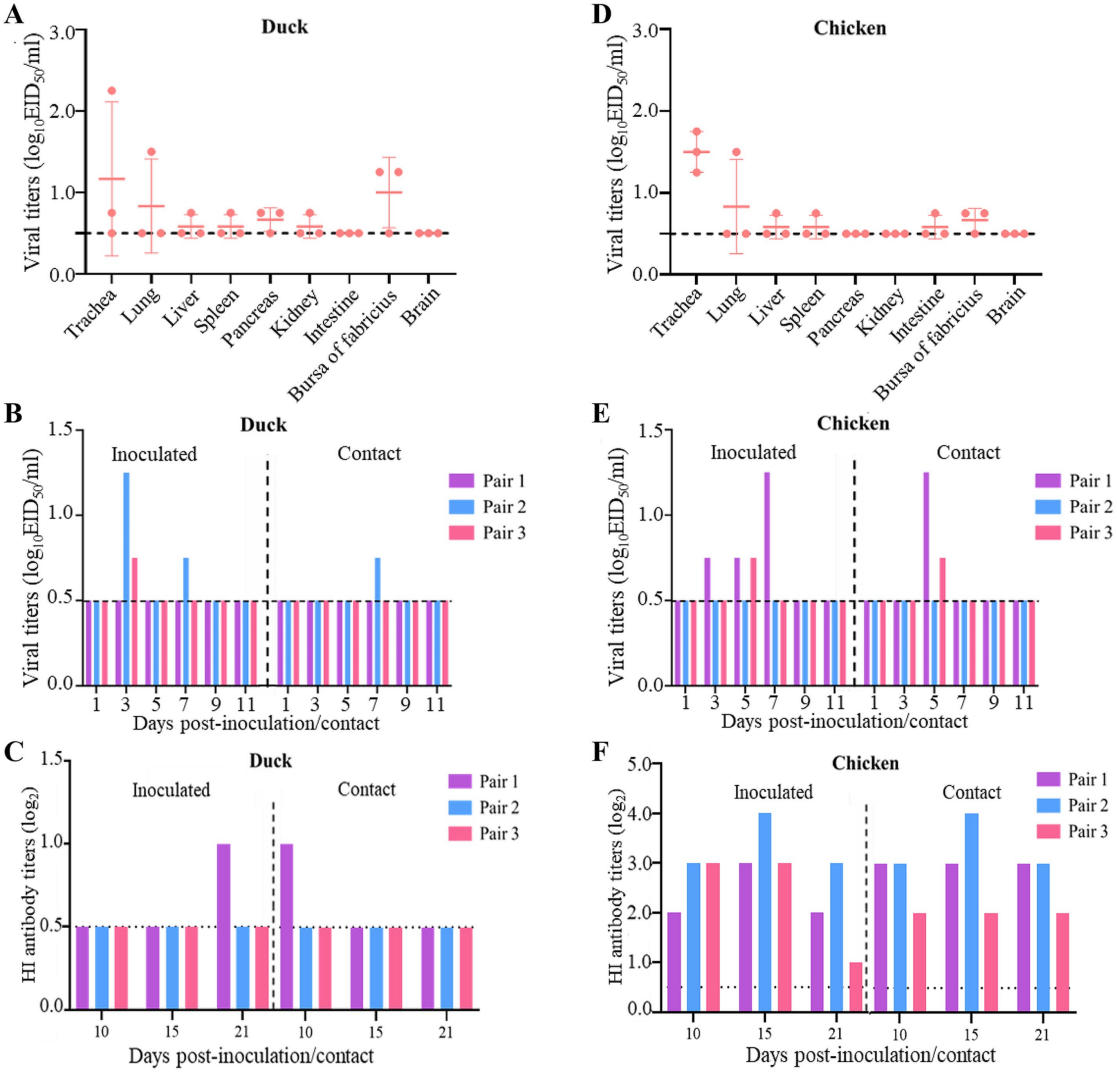
Replication and transmission of SX/2143 (H12N2) in ducks and chickens. **(A)** The replication of SX/2143 in ducks is shown. The organs were collected 3 days post infection, and the viruses were titrated in eggs. **(B)** The viral shedding of SX/2143 in inoculated and contact ducks is shown. **(C)** HI antibody is detected in the serum of the ducks. **(D)** The replication of SX/2143 in chickens is shown. The organs were collected 3 days post infection, and the viruses were titrated in eggs. **(E)** The viral shedding of SX/2143 in inoculated and contact chickens is shown. **(F)** HI antibody is detected in the serum of the chickens. The dashed lines indicate the lower limit of detection.

Wild birds and waterfowl are the primary natural hosts of the H12N2 virus. However, the replication capacity and horizontal transmission potential of the H12N2 virus in ducks and chickens remain to be thoroughly investigated. We initially assessed the replication capacity of the whooper swan-derived H12N2 virus in ducks and evaluated its transmission dynamics. The SX/2143 virus did not cause mortality or noticeable clinical symptoms in ducks throughout the observation period. The virus was detected in the trachea, pancreas, and bursa of Fabricius in two inoculated ducks, with viral titers ranging from 0.75 to 2.25 log_10_ EID_50_/ml (Figure 4A). Additionally, the virus was detected in the lung, liver, spleen, and kidney of one of the three inoculated ducks, with titers ranging from 0.75 to 1.5 log_10_ EID_50_/ml. No virus was detected in the intestine or brain of the inoculated ducks (Figure 4A). In transmission experiments, oropharyngeal and cloacal swabs were collected into the same tube and titrated in chicken embryos, revealing that the virus was detected in two of the three inoculated ducks on days 3 and 7 p.i., with viral titers ranging from 0.75 to 1.25 log_10_ EID_50_/ml (Figure 4B). The virus was also detected in one duck from the contact group, with a viral titer of 0.75 log_10_ EID_50_/ml on day 7 post-contact (p.c.) (Figure 4B). Serum samples were collected from both the inoculated and contact groups of ducks on days 10, 15, and 21 p.i. HI tests were performed to determine the serum antibody titers. The results indicated that one duck from the inoculated group and one duck from the contact group exhibited seroconversion, with antibody titers reaching 1 log2 (Figure 4C). The infection and transmission experiments in ducks demonstrated that while the H12N2 virus can infect domestic ducks, it replicates poorly and shows limited potential for contact transmission.

We further assessed the ability of this whooper swan-derived H12N2 virus to infect and transmit in chickens. As shown in Figure 4D, the virus was detected in the trachea of all three inoculated chickens, with viral titers ranging from 1.25 to 1.75 log_10_ EID_50_/ml. Low viral titers were also detected in the lung, liver, spleen, bursa of Fabricius, and intestine, whereas no virus was detected in the pancreas, kidney, or brain. The results from the transmission study indicated that the virus was detected in swab samples from two of three chickens in both the inoculated and contact groups, with viral titers ranging from 0.75 to 1.5 log_10_ EID_50_/ml (Figure 4E). Notably, the HI assay revealed that the sera from all inoculated and contact chickens tested positive for the H12N2 virus, with titers ranging from 1 log2 to 4 log2 on days 10, 15, and 21 p.i. (Figure 4F). In summary, the H12N2 virus derived from the whooper swan can replicate at low levels in both ducks and chickens, primarily within the respiratory system, including the lungs and trachea. In ducks, the H12N2 virus exhibited moderate transmission efficiency, with two out of three ducks showing evidence of transmission. However, in chickens, the virus has a high capacity for transmission, suggesting that the H12N2 virus may be better adapted to chickens than to ducks.

### 3.6 H12N2 virus can replicate in mice without prior adaptation

**Figure 5:**
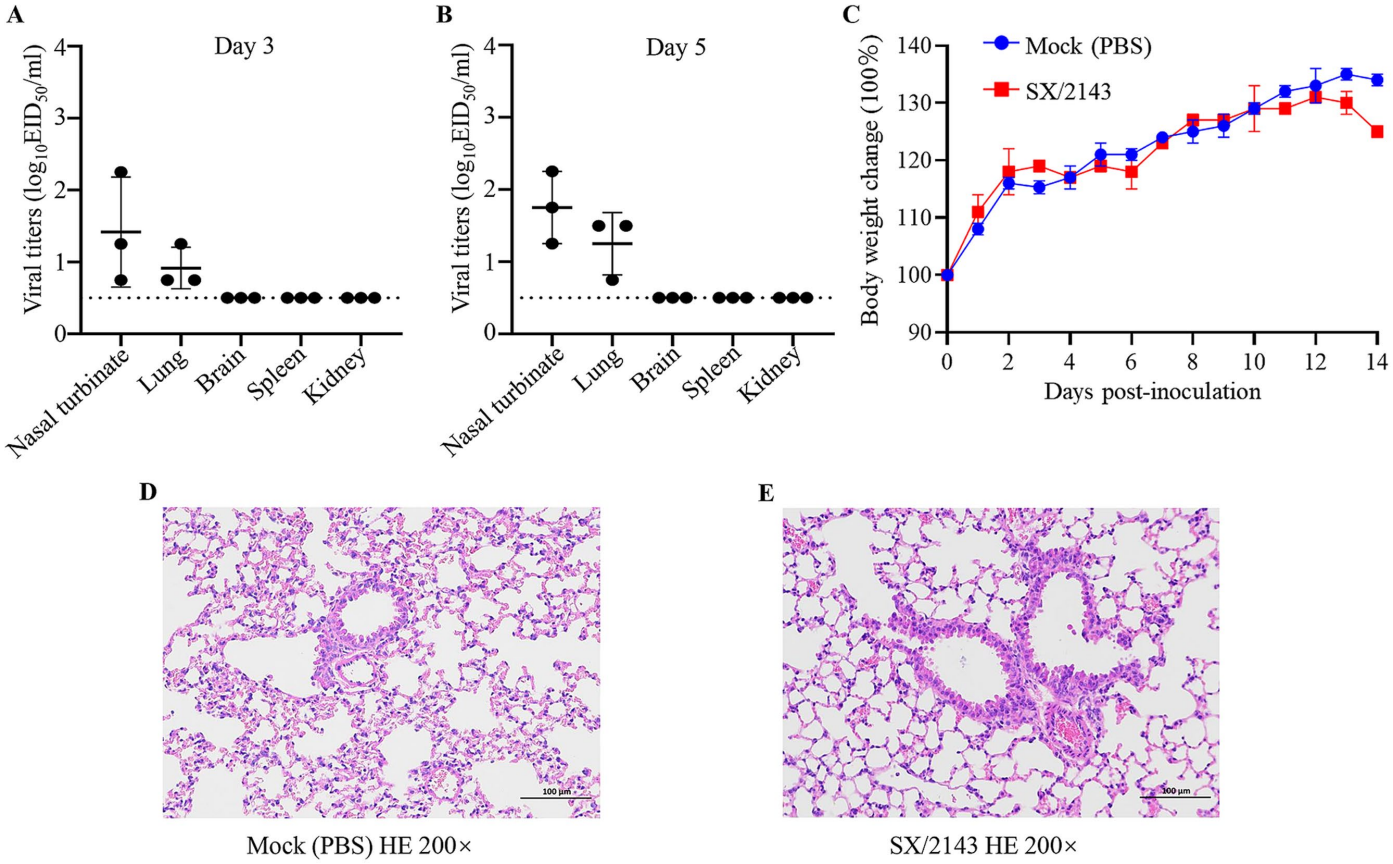
Replication of SX/2143 (H12N2) in mice. The nasal turbinate, lungs, brain, spleen, and kidney were collected on days 3 **(A)** and 5 **(B)** post infection and titrated in eggs. The dashed lines indicate the lower limit of detection. **(C)** Changes in the body weights of the mice during the observation period are shown. **(D)** H&E staining of lung tissues from mice inoculated with PBS is shown. **(E)** Pathological lung lesions are observed in mice inoculated with SX/2143, as shown by H&E staining. Images of pathological staining were captured at ×200 magnification.

Currently, there is a lack of relevant reports regarding the replication ability and pathogenicity of the H12N2 virus in mammals. To evaluate the potential infection risk of this wild bird-derived H12N2 virus in mammals, we conducted a study using a mouse model. BALB/c mice were inoculated with the SX/2143 virus at a dose of 10^6^ EID_50_, and organs were collected for viral titration on days 3 and 5 p.i. As shown in Figure 5, on days 3 and 5 p.i., the virus was detected in the nasal turbinate and lungs of the mice, with viral titers ranging from 0.75 to 2.25 log_10_EID_50_/ml (Figures 5A,B). No virus was detected in the spleen, kidneys, or brain. During the 14-day observation period, the mice inoculated with the H12N2 virus did not exhibit significant body weight loss, and no apparent clinical signs were observed (Figure 5C). Mild to moderate pathological lesions were observed in the lung tissue through hematoxylin and eosin (HE) staining (Figures 5D,E). These results suggest that the wild bird-derived H12N2 virus can replicate in the lungs and nasal turbinates of mice without prior adaptation.

### 3.7 Replication capacity of the H12N2 virus in MDCK and DF1 cells

**Figure 6:**
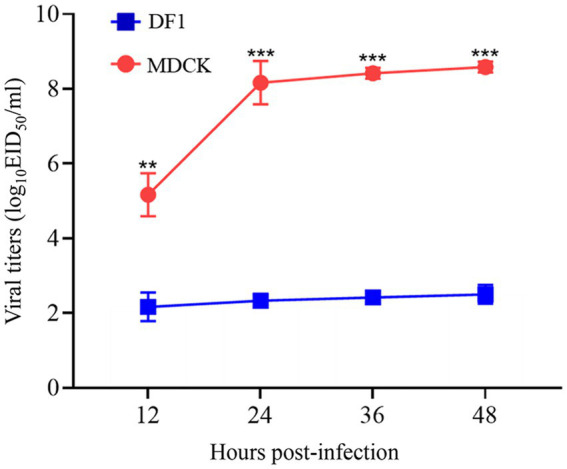
Growth curves of the SX/2143 (H12N2) virus in MDCK and DF1 cells. MDCK and DF1 cells were infected with SX/2143 at an EID_50_ of 10^4^. The cell supernatant was collected at the indicated times post infection and titrated in eggs. Viral titers are presented as the means ± SDs from three independent experiments. **, *p* < 0.01; ***, *p* < 0.001 (multiple t tests in GraphPad Prism 8).

To evaluate the replication capacity of the H12N2 virus at the cellular level *in vitro*, we conducted experiments using MDCK cells and DF1 cells, which are commonly used in influenza virus research. Following infection of MDCK or DF1 cells with the SX/2143 (H12N2) virus, supernatants were collected at 12, 24, 36, and 48 h.p.i. for viral titration. The results indicated that the virus exhibited poorer replication capacity in DF1 cells, and the viral titer increased from 2.17 log_10_ EID_50_/ml to 2.5 log_10_ EID_50_/ml from 12 to 48 h.p.i. (Figure 6). Notably, the H12N2 virus replicates efficiently in MDCK cells, with viral titers at various time points significantly higher than those in DF1 cells. In MDCK cells, the viral titer was 5.17 log_10_ EID_50_/ml at 12 h.p.i. and increased to 8.58 log_10_ EID_50_/ml at 48 h.p.i. (Figure 6). These results indicated that the H12N2 virus replicates efficiently in MDCK cells, suggesting that the virus has the potential for cross-species transmission to infect mammals.

## 4 Discussion

In recent years, humanity has faced threats from various emerging and re-emerging infectious diseases, one of which is the influenza virus (Jiang et al., 2024). Notably, there have been incidents of interspecies transmission of influenza viruses, such as H7N9, H3N8, and H10N3, that have directly infected humans. Genetic sequence analysis revealed that some gene fragments of these viruses originate from wild birds, indicating that they are recombinant viruses resulting from the reassortment of wild bird-derived viruses and poultry influenza viruses (He et al., 2023; Tian et al., 2023; Yu et al., 2023). Several studies have reported the isolation and identification of highly pathogenic avian influenza (HPAI) H5Nx viruses in whooper swans (Ke et al., 2022; Li et al., 2021; Li et al., 2020). The long-distance spread of these viruses during swan migration is considered one of the key factors contributing to global outbreaks of highly pathogenic H5Nx viruses. These HPAI H5 viruses have caused mortality events in poultry, wild birds, and wild mammals. In March 2024, the H5N1 clade 2.3.4.4b was confirmed in a dairy cow in the United States, and it has been demonstrated that this virus can infect cattle, spread between herds, and infect humans (Caserta et al., 2024; Gu et al., 2024).

The number of H12 influenza virus isolates reported is currently very limited. As of November 22, 2024, the total number of H12 virus isolates recorded in the global influenza virus public database was only 516, which was significantly lower than that of other common subtypes. Among these, there were only four isolates from swans, including one H12N3 isolate, two H12N1 isolates, and one isolate from this study. The H12 subtype exhibits different combination preferences with various NA subtypes, with H12N5 being the most prevalent, accounting for approximately 60% (Wang et al., 2024). In this study, a novel H12N2 reassortant was isolated from the fresh feces of migratory swans, and its biological characteristics were evaluated. Although previous studies have demonstrated reassortment of H12 viruses between the North American lineage and Eurasian lineage (Sharshov et al., 2019), most H12 viruses isolated from North American and Eurasian samples are genetically distinct. This genetic divergence has led to the formation of two separate lineages in the phylogenetic tree, and the HA and NA genes of the SX/2143 strain were both from the Eurasian lineage (Figures 2, 3). Interestingly, the N2 gene of this H12N2 virus may have originated from the N2 gene of H9N2 viruses. We speculate that this could be one of the reasons for the better adaptability of the virus to poultry and mammals. Analysis of the genetic evolution of the internal genes revealed that the six internal genes of SX/2143 originated from other subtypes, such as H3, H2, and H8, which were isolated from wild birds or waterfowl (Supplementary Figure S1). These findings indicate that the SX/2143 virus is a complex recombinant strain, highlighting the importance of the ongoing epidemiological surveillance of AIVs in wild birds to enhance the understanding of the evolutionary trends of influenza viruses in a timely manner and to promptly identify new genotypes.

Previous studies have shown that AIVs preferentially bind to *α*-2,3-linked sialic acids (SAs), whereas human isolates preferentially bind to α-2,6-linked SAs (Rogers and Paulson, 1983). Mutations in the receptor-binding domain of HA enable AIVs to possess human-type receptor-binding characteristics, which are among the important factors contributing to the cross-species transmission capability of AIVs (Herfst et al., 2020). Amino acids at positions Q226L and G228S of the HA protein are critical for receptor binding transitions in AIVs (Vines et al., 1998). The SX/2143 virus has the common residues Q226 and G228 of AIVs, suggesting that this virus may still retain avian-type receptor-binding characteristics. Mutations at certain key amino acid sites have been shown to be associated with the pathogenicity or transmissibility of AIVs (Ayllon et al., 2014; Elgendy et al., 2017; Feng et al., 2016; Guo et al., 2022; Hulse-Post et al., 2007). In this study, we found that the SX/2143 virus harbors several amino acid mutations in PB1, PA, and other proteins that are associated with increased pathogenicity, suggesting a potential increase in replication ability and virulence in mammals (Table 1). A previous study of wild duck-origin H12N5 indicated that the H12N5 virus could replicate at low levels in ducks and chickens and transmit with low efficiency (although low levels of viral shedding were detected, no HI antibodies were detected in ducks or chickens in the transmission groups) (Wang et al., 2024). In this study, the H12N2 virus showed similar results, as it could replicate and transmit at low levels in ducks. However, notably, the virus was able to be transmitted efficiently in chickens, suggesting better adaptation of the virus to chickens (Figure 4). We speculate that this may be related to the N2 gene of the H12N2 virus originating from H9N2, as well as the presence of certain key amino acid mutations in its internal genes (such as D3V and D622G in the PB1 gene), which require further investigation. Unlike previous studies, in which H12 viruses could not be detected in the lungs of mice without prior adaptation (Wan et al., 2022; Wang et al., 2024), the H12N2 virus in this study was detectable in both the lungs and nasal turbinates of mice (Figure 5). Furthermore, we evaluated the replication ability of the H12N2 virus at the cellular level *in vitro* and found that the virus replicated efficiently in MDCK cells (Figure 6), suggesting a potential risk of infection in mammals.

In summary, we isolated a novel H12N2 virus from whooper swans in Central China, conducted a systematic phylogenetic analysis, and evaluated its replication ability in animals and at the cellular level. The H12N2 virus was found to replicate in mice and replicate efficiently in mammalian cells, suggesting a potential risk of mammalian infection. These findings highlight the need to strengthen epidemiological surveillance and risk assessment of AIVs originating from wild birds.

## Data Availability

The datasets presented in this study can be found in online repositories. The names of the repository/repositories and accession number(s) can be found in the article/Supplementary material.
