# Supplementary material for: Phylogeography and biological characterization of H12N2 virus isolated from whooper swan in Central China
Source: Front Microbiol. 2025 Jan 9;15:1536876. doi: 10.3389/fmicb.2024.1536876 (PMC11754294; doi:10.3389/fmicb.2024.1536876)
Supplement: Supplementary file 1 [file Data_Sheet_1.docx]

Supplementary Material

**Phylogeography and biological characterization of H12N2 virus isolated from whooper swan in Central China**

**Pengfei Ren****^1, †^, Zhen Gao^1, †^, Xing Li^1^, Jiao Tang^1^, Pei Li^1^, Zhonglin Huang^1^, Jinchi Guo^1^, Pengfei Cui^2^, Lin Jin^1, *^, Junping Li^1, *^, Libin Liang^1, *^**

^1^College of Veterinary Medicine, Shanxi Agricultural University, Jinzhong, China

^2^State Key Laboratory for Animal Disease Control and Prevention, Harbin Veterinary Research Institute, Chinese Academy of Agricultural Sciences, Harbin, China

*** Correspondence**

Lin Jin, jinlin2021@sxau.edu.cn

Junping Li, lijunping@sxau.edu.cn

Libin Liang, lianglibin@sxau.edu.cn

**^†^** These authors contributed equally to this work.

**Supplementary Table 1.** Genetic similarity of the SX/2143 (H12N2) virus in this study with the most related avian influenza viruses.

| Gene | The highest nucleotide homologous strains in GISAID | Nucleotide identity | Accession numbers |
| --- | --- | --- | --- |
| PB2 | A/environment/Kagoshima/KU-J3/2022 (H3N8) | 99.34% | EPI2789467 |
| PB1 | A/gadwall/Novosibirsk region/982k/2018 (H3N8) | 98.90% | EPI1352460 |
| PA | A/Mallard/South Korea/KNU2021-52/2021(H8N4) | 99.26% | EPI2153587 |
| HA | A/duck/Kaohsiung/20WB0201-52/2020 (H12N5) | 98.23% | EPI3407491 |
| NP | A/duck/Bangladesh/WF-433/2024 (H8) | 99.53% | EPI3354456 |
| NA | A/duck/Bangladesh/19D2183/2023 (H9N2) | 98.44% | EPI3116102 |
| M | A/mallard/Beijing/10/2016 (H4N6) | 99.59% | EPI1074786 |
| NS | A/Mallard/Korea/KNU25/2023(H1N1) | 100.00% | EPI2873293 |

##
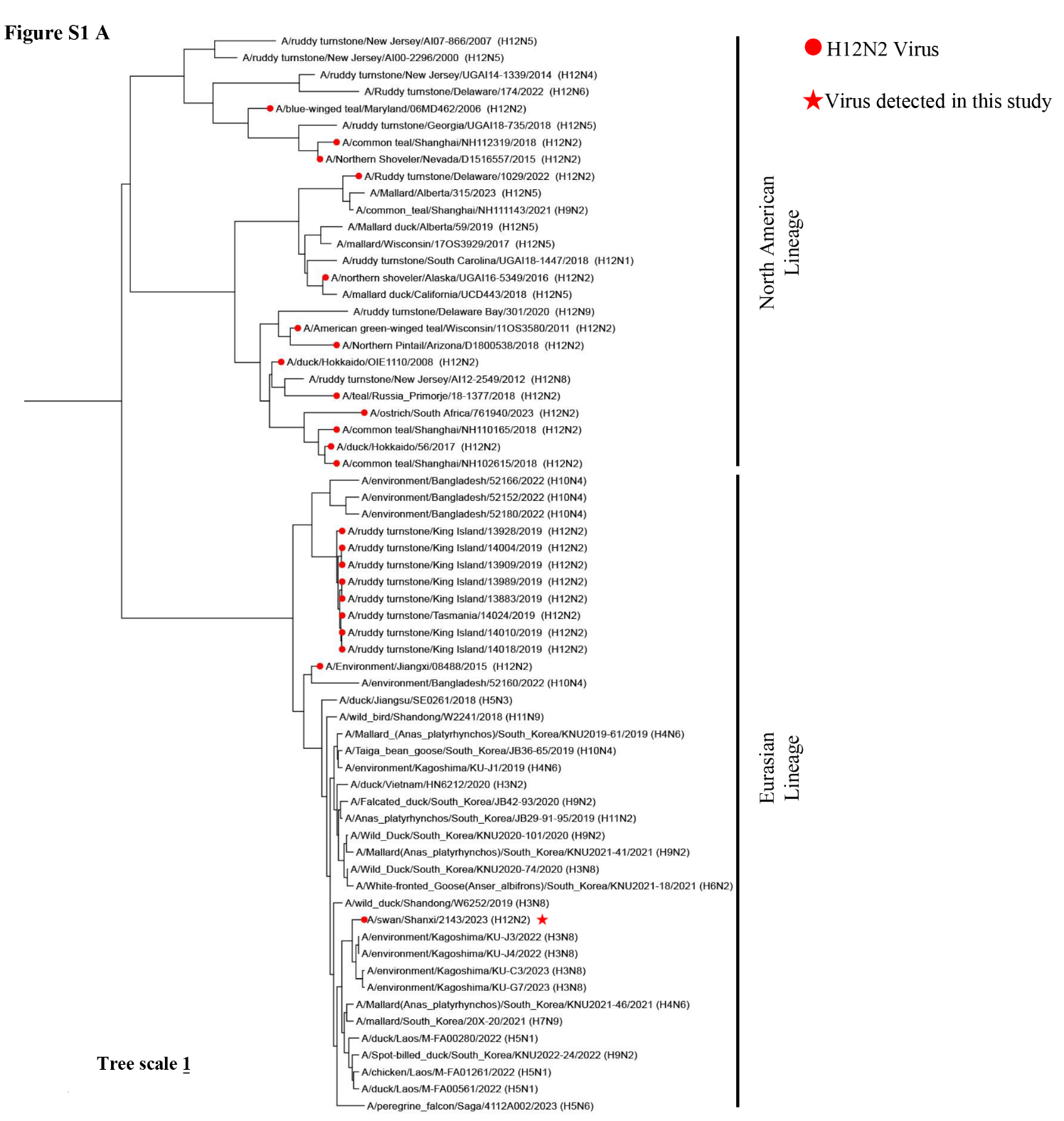
Supplementary Figures


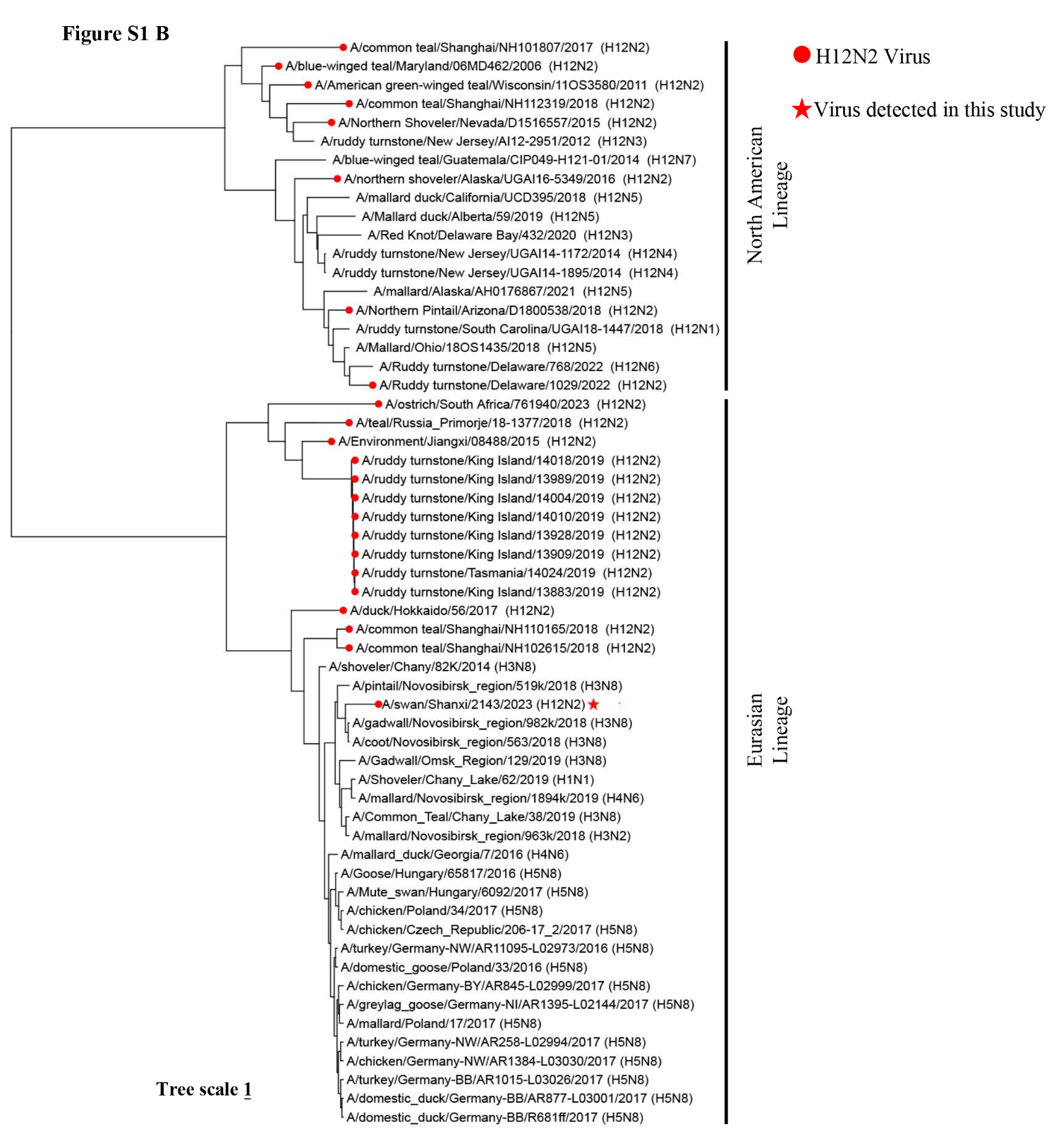


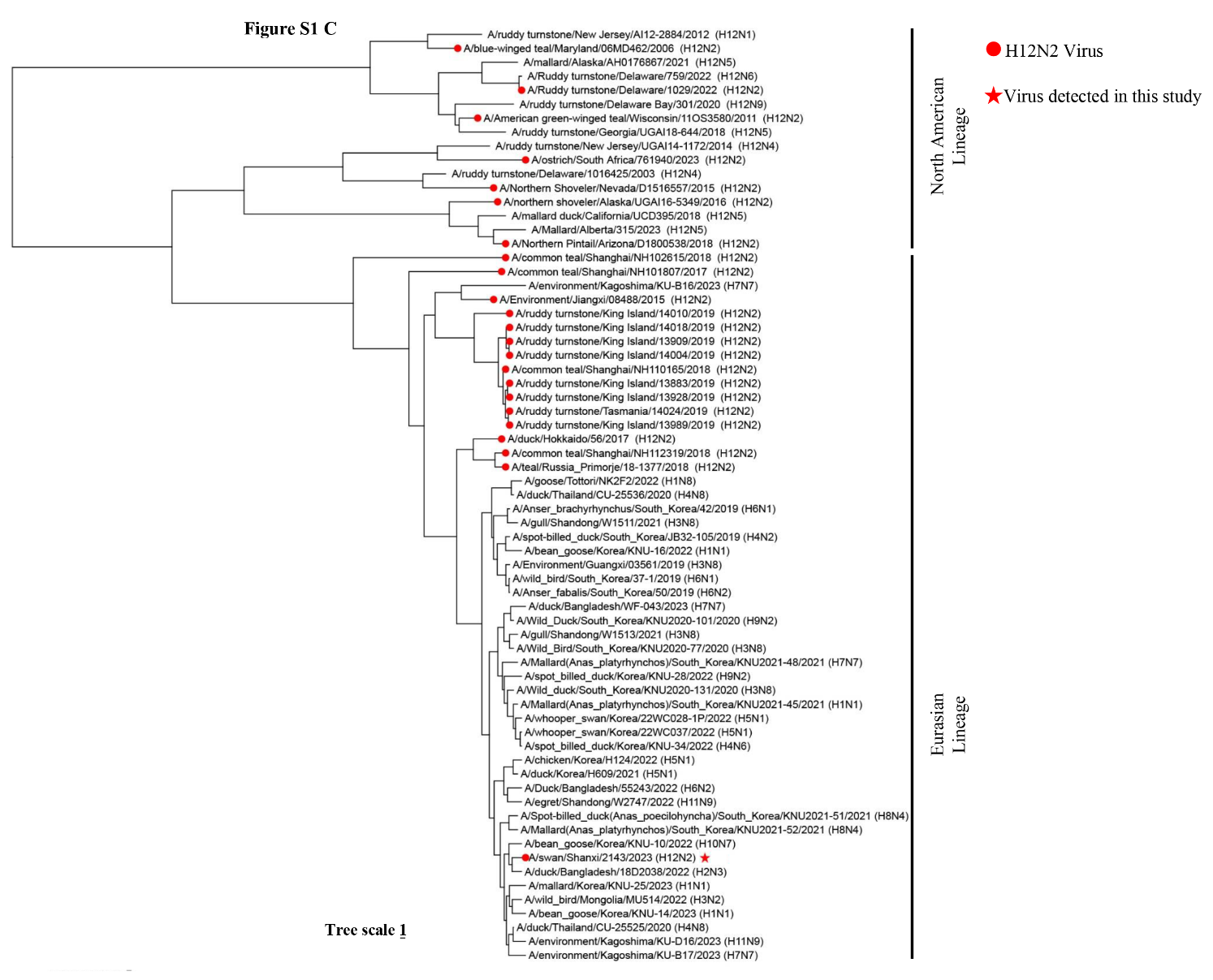


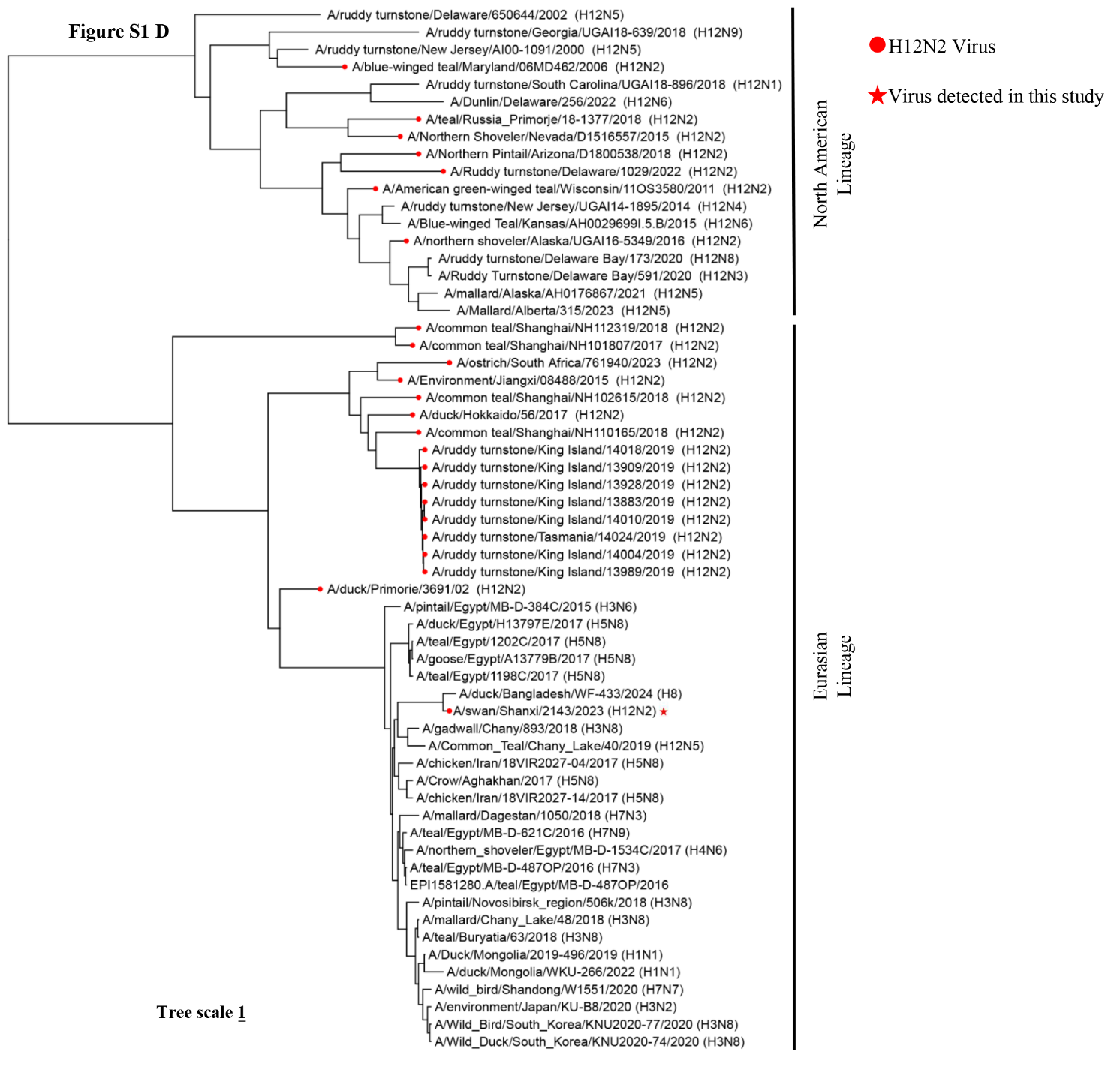


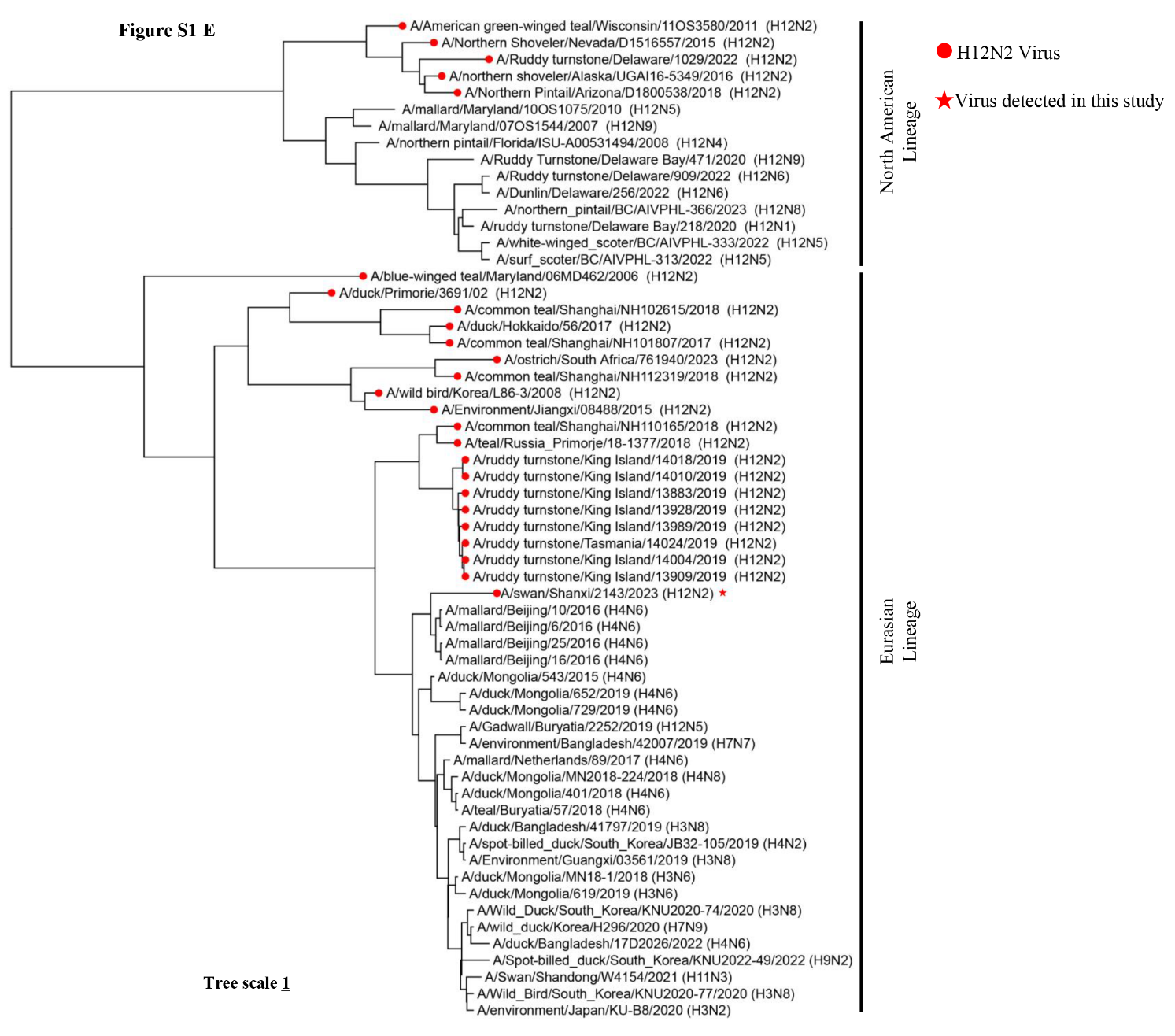


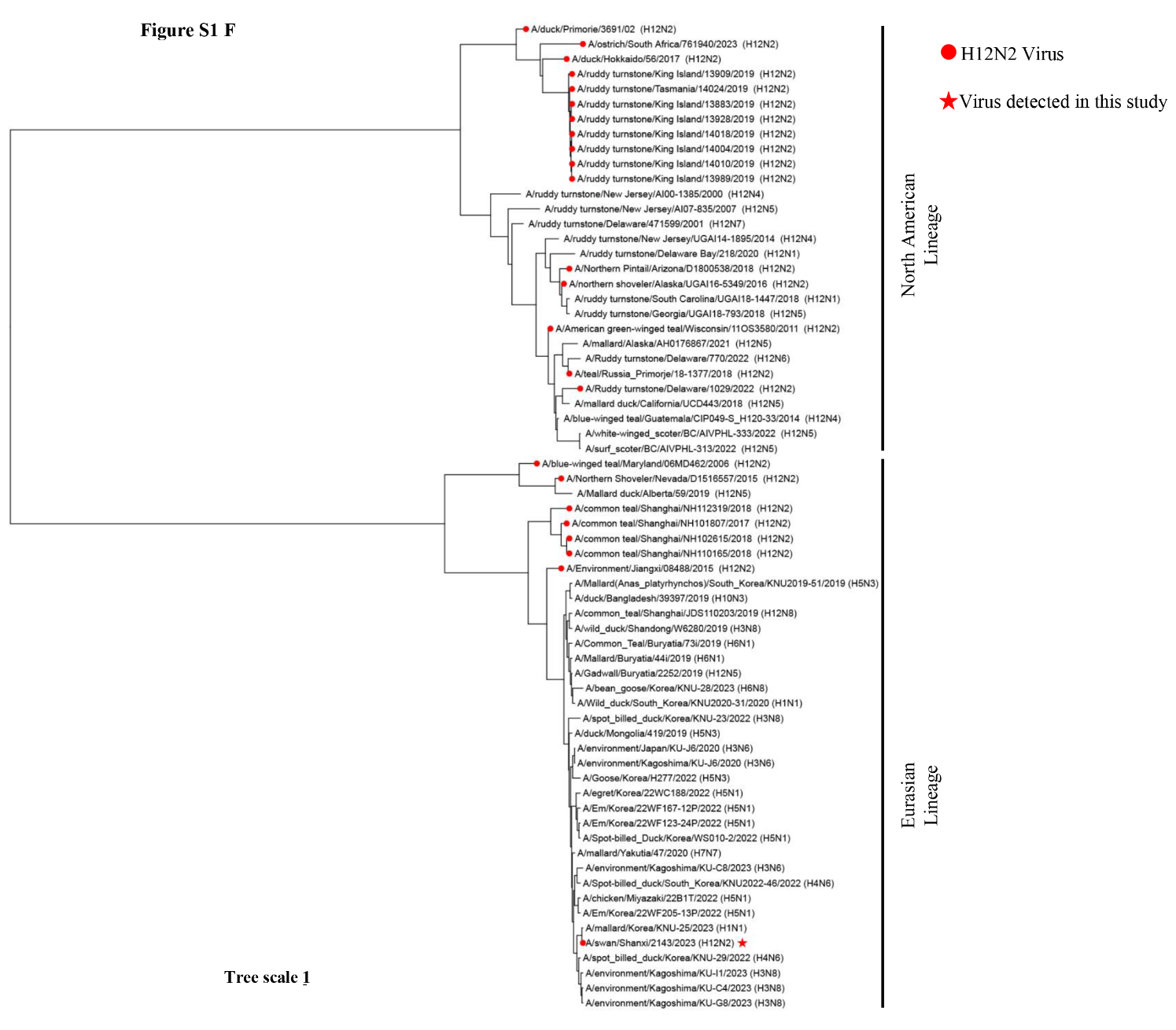


**Supplementary Figure 1.** Phylogenetic trees of PB2 (A), PB1 (B), PA (C), NP (D), M (E), and NS (F) genes of H12N2 viruses. Red circles indicate H12N2 viruses and red stars indicate virus detected in this study.
